# Coagulase gene polymorphisms of *Staphylococcus aureus* isolates from patients at Kosti Teaching Hospital, Sudan

**DOI:** 10.1099/acmi.0.000026

**Published:** 2019-05-15

**Authors:** Omer Mohammed Ali Ibrahim, Naser Eldin Bilal, Mohammed E. H. Azoz, Hanan B. Eltahir

**Affiliations:** 1 Department of Microbiology, Faculty of Medical Laboratory Sciences, University of El-Imam El-Mahdi, Kosti, Sudan; 2 Department of Medical Microbiology, Faculty of Medical Laboratory Sciences, University of Khartoum, Khartoum, Sudan; 3 Department of Surgery, Faculty of Medicine, University of El-Imam El-Mahdi, Kosti, Sudan; 4 Department of Biochemistry, Faculty of Medicine, University of El-Imam El-Mahdi, Kosti, Sudan

**Keywords:** *Staphylococcus aureus*, methicillin-resistant, PCR-RFLP, *coagulase* gene polymorphism

## Abstract

**Background:**

*
Staphylococcus aureus* is a common cause of nosocomial infections. Epidemiological typing of *S. aureus* enables control of its spread. The objective of this study was to investigate coagulase gene polymorphisms of *S. aureus* isolated from patients at Kosti Hospital in Sudan.

**Methods:**

In total, 110 *S. aureus* isolates were recovered from 110 patients who were enrolled in the study. *S. aureus* strains were isolated on blood agar and MacConkey agar and then identified by conventional tests. Resistance to methicillin was determined by detection of the *mecA* gene. Polymorphism in the coagulase gene (*coa*) was investigated using PCR followed by *Alu*I RFLP analysis.

**Results:**

Methicillin-resistant *S. aureus* accounted for 62/110 (56 %) of the isolates. PCR of the *coa* gene showed two different amplicons, one of 680 bp detected in 83/110 (75.5 %) of the isolates and one of 790 bp detected in 27/110 (24.5 %). When digested with the *Alu*I enzyme, the 790 bp amplicon resulted in three restriction fragments of 500, 210 and 80 bp (*coa*1). Restriction of the 680 bp amplicon gave two patterns; the first (*coa*2) was found in 22/110 (20 %) of the isolates and showed four fragments of 210, 210, 180 and 80 bp, and the second (*coa*3) was found in 61/110 (55.5 %) and revealed three fragments of 390, 210 and 80 bp. Most of the *coa*3 isolates (75.4%) were methicillin-resistant.

**Conclusion:**

Three polymorphic genotypes of *S. aureus* were identified in patients at Kosti Hospital. The *coa*3 genotype was the predominant one and was mostly detected in methicillin-resistant isolates.

## Introduction

*Staphylococcus aureus* strains are a major cause of human infections. They have many virulence factors that enable them to cause disease even in normal hosts. Antibiotic resistance is a common feature among *S. aureus* strains. Methicillin resistance in staphylococci is primarily due to acquisition of the *mecA* gene [1]. The *mecA* gene encodes an altered penicillin-binding protein designated as PBP2a [2]. The affinity of beta-lactam towards PBP2a is much lower than towards native PBP. Thus, expression of this protein leads to resistance to all beta-lactam antibiotics, including methicillin [3]. Therefore, the method of choice for detection of methicillin-resistant *S. aureus* (MRSA) is PCR through detection of the *mecA* gene [4].

Nasal carriage of *S. aureus* has been identified as a major risk factor in the development of infections both in hospitals [5] and in the community [6]. Monitoring the spread of *S. aureus* strains requires efficient epidemiological typing systems that allow the discrimination between unrelated isolates. The typing system should be rapid, low cost and easy to interpret [7]. Phenotypic typing methods such as phage typing and antimicrobial susceptibility testing have long been used for the discrimination of *S. aureus* strains [8]. Phenotypic typing methods, however, do not directly characterize the expression of different genes. Some of the methods show low typeability and discriminatory power [9]. Genotypic typing methods are based on the analysis of chromosomal or extrachromosomal DNA. Plasmid analysis was the first DNA-based method to be applied to *S. aureus* [10]. RFLP analysis with a variety of DNA and RNA probes has also been used to type bacterial strains [11]. Several PCR-based methods have been developed, in which specific genes with variable repeat regions, such as coagulase (*coa*) and *spa* [12], or polymorphic non-coding repetitive sequences dispersed around bacterial genomes, serve as targets for PCR amplification [13].

Coagulase is an enzyme produced by *S. aureus* that clots plasma. It binds to prothrombin; together they become enzymatically active and initiate fibrin polymerization. The enzyme may deposit fibrin on the surface of *S. aureus* and perhaps prevent phagocytosis or its destruction within phagocytic cells [14]. Coagulase gene amplificons are highly polymorphic as a result of differences in sequence at the 3′ variable region that lead to high discriminatory power that could be further discriminated by digestion with a restriction enzyme [15], such as *Alu*I. PCR amplification of the coagulase gene followed by digestion with an endonuclease enzyme is considered a simple, rapid and accurate typing method that can be included in infection control programmes and epidemiological studies [16]. The objective of this study was to investigate coagulase gene polymorphisms among *S. aureus* isolated from patients at Kosti Teaching Hospital in Sudan.

## Methods

### *Staphylococcus aureus* isolation and identification

A cross-sectional study was conducted at Kosti Teaching Hospital from May 2013 to July 2014. In total, 110 *S. aureus* isolates were collected from a wound or nasal cavity from 110 patients (one isolate from each patient) who attended the surgery department. All specimens were inoculated onto blood agar and MacConkey agar media then incubated aerobically for 24 h at 37 °C. The appearance of golden-yellow colonies on blood agar and pink lactose-fermenting colonies on MacConkey agar was presumed to indicate *S. aureus *, which were then identified using a Gram staining technique. Colonies that showed Gram-positive cocci arranged in clusters under the microscope were purified on nutrient agar and subjected to conventional biochemical tests, i.e. catalase, coagulase tube, DNase test agar and mannitol fermentation (Mannitol Salt Agar) tests [17]. Catalase-positive, coagulase-positive, DNase-positive and mannitol-fermenting isolates were considered *S. aureus* and then frozen at −20 °C. Preservation was done from a single representative colony of pure growth of *S. aureus *, by inoculation of 1.5 ml tryptic soy broth containing 15 % glycerol, in screw-capped cryovials, incubated overnight at 37 °C before storing at −20 °C.

### Molecular analysis

The isolates were considered MRSA or methicillin-susceptible *S. aureus* (MSSA) depending on the presence or absence of the *mecA* gene, respectively. In addition to the biochemical tests, the presence of the coagulase gene confirmed that these isolates were *S. aureus *. All isolates were characterized by PCR amplification followed by RFLP analysis of their coagulase gene (*coa*) using the *Alu*I restriction enzyme.

### Bacterial DNA isolation

All preserved isolates were re-identified as described previously [17]. Pure cultures were grown on nutrient agar. A representative colony was touched by the wire loop; the gathering inoculum was aseptically transferred and emulsified into 1.5 ml tryptic soy broth in a sterile Eppendorf tube and incubated overnight at 37 °C. The overnight bacterial growth was centrifuged and the sediment was used for DNA extraction. DNA was extracted from bacterial cells using a G-spin Genomic DNA Extraction Kit according to the manufacturer's instructions. The extracted DNA was preserved at −20 °C.

### PCR-RFLP assays

PCR was done using iNtRON's Maxime PCR PreMix master mix, and the *mecA* gene forward primer 5′AAAATCGATGGTAAAGGTTGGC3′ and reverse primer 5′AGTTCTGC AGTACCGGATTTGC3′ were selected on the basis of the published nucleotide sequence and used for detection of MRSA [18]. The components of the PCR mix were dissolved in distilled water to a total volume of 20 µl including 2 µl of the DNA and 2 µl (20 pmol) for each of the primers. After an initial denaturation at 94 °C for 45 s, the cycling PCR machine continued for 30 cycles of denaturing at 94 °C for 20 s, annealing at 57 °C for 15 s and extension at 70 °C for 15 s extension, with a final step at 72 °C for 2 min. The PCR product was then analysed by electrophoresis on a 1.5 % agarose gel stained with ethidium bromide using Tris-borate-EDTA (1XTBE) as running buffer [19]. Coagulase gene amplification was done using iNtRON's Maxime PCR PreMix master mix and 75 pmol (0.75 µl) for each of the primers. The forward primer 5′ATAGAGATGCTGGTACAGG3′ and the reverse primer 5′GCTTCCGATTGTTCGATGC3′ were selected according to the published nucleotide sequences. The procedure, cycle programme and electrophoresis were similar to those used for the *mecA* gene [19].

Approximately 15 µl of coagulase PCR product was digested with 4 U of restriction endonuclease *Alu*I, at 37 °C for 1 h. Ten microlitres of digested PCR product was analysed by electrophoresis on 2% agarose. Approximate sizes of the amplicons and restriction fragment were estimated based on the agarose gel electrophoresis profile.

A standard strain of *S. aureus ,* ATCC 25923 (coagulase gene-positive and *mecA* gene-negative), was used as a positive control for the coagulase gene and negative control for the *mecA* gene. A clinical strain identified by conventional biochemical methods as *Staphylococcus saprophyticus* (coagulase-negative) [17] was used as a negative control. A clinical *S. aureus* strain that was positive in an MRSA-latex agglutination test of PBP2 and that was *mecA* gene-positive was included as a positive control for the *mecA* gene.

## Results

In total, 110 *S. aureus* isolates were collected from 110 hospitalized patients [67 (60.9 %) males and 43 (39.1 %) females]. Sixty-one (55.5%) of the isolates were from nasal swabs and 49 (44.5 %) were from wound infection from different sites of the body. PCR amplification of the *mecA* gene revealed that 62/110 (56.4 %) of the isolates were *mecA* gene carriers (MRSA) and 48/110 (43.6 %) were not (MSSA).

**Fig. 1.**
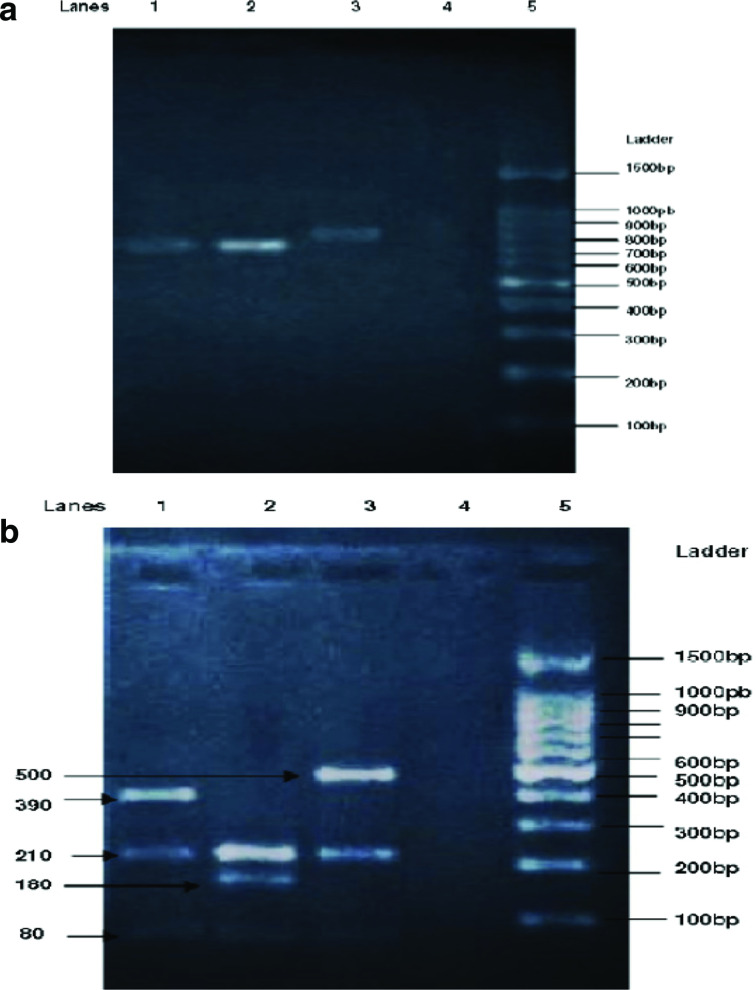
PCR-RFLP analysis of the *S. aureus* *coa* gene (representative isolates). (a) Coagulase gene PCR product electrophoresis on 1.5 % agarose gel. Lanes 1 and 2, coagulase PCR product of 680 bp; lane 3, coagulase PCR product of 790 bp; lane 4, negative control; lane 5, ladder. (b) Coagulase gene PCR-RFLP restricted by *Alu*I, electrophoresis on 2% agarose gel. Lane 1, coagulase 680 bp fragmented into three (390, 210 and 80 bp); lane 2, coagulase 680 bp fragmented into four (210, 210, 180 and 80 bp); lane 3, cut of 790 bp into three (500, 210 and 80 bp) fragments; lane 4, negative control; lane 5, ladder.

PCR-RFLP genotyping of *S. aureus* based on amplification of the coagulase gene (*coa*) followed by digestion with the *Alu*I enzyme showed two different amplicons, one of 680 bp detected in 83/110 (75.5 %) of the isolates and one of 790 bp detected in 27/110 (24.5 %). When digested with the *Alu*I enzyme, the 790 bp amplicon was restricted into three different fragments of 500, 210 and 80 bp which represents restriction pattern 1 (*coa*1). The 680 bp product was restricted into two patterns. The first pattern represents restriction pattern 2 (*coa*2) found in 22/110 (20 %) of the total isolates, and showed four different fragments of 210, 210, 180 and 80 bp. Restriction of the amplicon repeatedly showed three bands with a total size of 470 bp, and we therefore concluded that there were two overlapping bands of 210 bp. The second pattern represents restriction pattern 3 (*coa*3) detected in 61/110 (55.5 %) of the isolates, and showed three different fragments of 390, 210 and 80 bp (Fig. 1). Most of the methicillin-resistant isolates, 46/62 (74.2 %), were from pattern 3 (*coa*3). However, most of the methicillin-susceptible isolates, 33/48 (68.7 %), were from pattern 1 (*coa*1) or pattern 2 (*coa*2) (Table 1). The results also showed that the most frequent genotype in interior nares was *coa*3, 32/61 (52.5 %), followed by *coa*1, 20/61 (32.8 % ), and *coa*2, 9/61 (14.7 % ). The most frequent genotype in wound infections was also *coa*3, 29/49 (59.2 %), followed by *coa*2, 13/49 (26.5 %), and then *coa*1, 7/49 (14.3 %).

**Table 1.** Comparison between methicillin-resistant (MRSA) and methicillin susceptible (MSSA) coagulase restriction fragment patterns due to endonuclease *Alu*I. Isolates were from 110 patients at Kosti Hospital [61 (55.5 %) from nasal swabs and 49 (44.5%) from wound swabs]

	Isolates	Coagulase length 790 bp	Coagulase length 680 bp		Total
		Pattern 1	Pattern 2	Pattern 3	
MSSA	Nasal carriage	15 (44.1 %)	7 (20.6 %)	12 (35.3 %)	34 (100 %)
	Wound infections	2 (14.3 %)	9 (64.3 %)	3 (21.4 %)	14 (100 %)
	Total	17 (35.4 %)	16 (33.3 %)	15 (31.3 %)	48 (100 %)
MRSA	Nasal carriage	5 (18.5 %)	2 (7.4 %)	20 (74.1 %)	27 (100 %)
	Wound infections	5 (14.3 %)	4 (11.4 %)	26 (74.3 %)	35 (100 %)
	Total	10 (16.1 %)	6 (9.7 %)	46 (74.2 %)	62 (100 %)
Total *S. aureus *	MSSA+MRSA	27 (24.5 %)	22 (20 %)	61 (55.5 %)	110 (100 %)

Pattern 1, three fragments: 500, 210 and 80 bp.

Pattern 2, four fragments: 210, 210, 180 and 80 bp.

Pattern 3, three fragments: 390, 210 and 80bp.

## Discussion

Typing of *S. aureus* is very important in the description of epidemiology and in infection control strategies. In this study at Kosti Teaching Hospital, PCR-RFLP of the coagulase gene showed two types of the coagulase amplicons (790 and 680 bp) and three fragments patterns of 500, 210 and 80 bp (pattern 1), 210, 210, 180 and 80 bp (pattern 2), and 390, 210 and 80 bp (pattern 3). These results were in disagreement with Bin Hameed [20] who reported in his study at Khartoum Teaching Hospital, Sudan, two coagulase amplicons (500 and 580 bp) that were restricted by *Alu*1 into two patterns each of two fragments, i.e. 190 and 310 bp, and 190 and 390 bp. However, the amplicon of 680 bp revealed in this study was in agreement with that reported by Sanjiv *et al.* [21], but with a different restriction pattern, with Sanjiv *et al.* reporting one pattern of three fragments of 210, 210 and 260 bp. Several studies showed variation in PCR-amplified coagulase gene sizes. Hookey *et al.* [19] reported four PCR-amplified coagulase gene sizes of 875, 660, 603 and 547 bp, the number of fragments produced upon *Alu*I digestion varied from one to four, and their sizes varied from 80 to 660 bp. Sanjiv *et al.* [21] detected three sizes of 600, 680 or 850 bp, and three *Alu*I restriction patterns of two or three fragments were obtained from PCR products with their sizes varying from 170 to 390 bp. Karakulska *et al.* [22] studied *S. aureus* strains isolated from cow's milk and reported that a PCR coagulase gene amplicon of 1030 bp was digested with *Alu*I into one restriction pattern of four fragments, of 470, 300, 170 and 90 bp. Such variation in results may reflect the discriminatory power of coagulase gene amplification [15].

Our study showed that the predominant *S. aureus* genotype in patients at Kosti Teaching Hospital was *coa*3, and 75.4 % of *coa*3 isolates were methicillin-resistant. It could be presumed that the majority of infections in particular regions are caused by *S. aureus* strains with certain *coa* genotypes [23]. Given its good discriminatory power, ease of use and cost effectiveness, RFLP of the *coa* gene can be used in epidemiological research to control and monitor hospital- and community-acquired *S. aureus* infections.

### Conclusion

PCR-RFLP genotyping of the coagulase gene detected three polymorphic forms of *S. aureus* (*coa1*, *coa2* and *coa3*) in patients at Kosti Teaching Hospital. The predominant genotype was *coa3* and 75.4 % of such isolates were methicillin-resistant. DNA sequence analysis could provide more specificity in the characterization of *S. aureus* polymorphisms and their evolutionary relationships.
